# Relationship among muscle strength, muscle endurance, and skeletal muscle oxygenation dynamics during ramp incremental cycle exercise

**DOI:** 10.1038/s41598-024-61529-x

**Published:** 2024-05-22

**Authors:** Shinji Nemoto, Tohru Nakabo, Naonori Tashiro, Asami Kishino, Akira Yoshikawa, Daisuke Nakamura, Eiichi Geshi

**Affiliations:** 1 Department of Physical Therapy, Showa University School of Nursing and Rehabilitation Sciences, 1865 Tokaichiba-cho, Midori-ku, Yokohama, Kanagawa 226-8555 Japan; 2 Division of Health Science Education, Showa University School of Nursing and Rehabilitation Sciences, Yokohama, Japan; 3 Office of Institutional Research, Showa University, Tokyo, Japan

**Keywords:** Cardiopulmonary exercise testing, Muscle endurance, Skeletal muscle oxygenation, Muscle oxygen saturation, Muscle strength, Near-infrared spectroscopy, Health care, Health occupations, Medical research

## Abstract

Peak oxygen uptake (VO_2_), evaluated as exercise tolerance, is a strong predictor of life prognosis regardless of health condition. Several previous studies have reported that peak VO_2_ is higher in those with a greater decrease in muscle oxygen saturation (SmO_2_) in the active muscles during incremental exercise. However, the skeletal muscle characteristics of individuals exhibiting a greater decrease in SmO_2_ during active muscle engagement in incremental exercise remain unclear. This study aimed to clarify the relationship among muscle strength, muscle endurance, and skeletal muscle oxygenation dynamics in active leg muscles during incremental exercise. Twenty-four healthy young men were included and categorized into the non-moderate-to-high muscular strength and endurance group (those with low leg muscle strength, endurance, or both; n = 11) and the moderate-to-high muscular strength and endurance group (those with both moderate-to-high leg muscle strength and endurance; n = 13). All participants underwent cardiopulmonary exercise testing combined with near-infrared spectroscopy to assess whole-body peak VO_2_ and the change in SmO_2_ at the lateral vastus lateralis from rest to each exercise stage as skeletal muscle oxygenation dynamics. A linear mixed-effects model, with the change in SmO_2_ from rest to each stage as the dependent variable, individual participants as random effects, and group and exercise load as fixed effects, revealed significant main effects for both group (*P* = 0.001) and exercise load (*P* < 0.001) as well as a significant interaction between the two factors (*P* < 0.001). Furthermore, multiple-comparison test results showed that the change in SmO_2_ from rest to 40%–100% peak VO_2_ was significantly higher in the moderate-to-high muscular strength and endurance group than in the non-moderate-to-high muscular strength and endurance group. Maintaining both muscle strength and endurance at moderate or higher levels contributes to high skeletal muscle oxygenation dynamics (i.e., greater decrease in SmO_2_) during moderate- or high-intensity exercise.

## Introduction

Exercise tolerance reflects overall endurance and is strongly influenced by levels of physical activity. Physical inactivity is closely associated with low exercise tolerance, a major risk factor for many chronic diseases that contributes to poor prognosis regardless of health condition^1–4^. Nonetheless, exercise tolerance is a modifiable factor that can be improved by increasing daily physical activity. Even small enhancements in exercise tolerance are associated with a reduced risk of all-cause mortality^5^. Therefore, improving exercise tolerance is crucial for maintaining good health.

Cardiopulmonary exercise testing (CPX) is the gold standard method for evaluating exercise tolerance^6–8^. In CPX, exercise tolerance, represented by peak oxygen uptake (VO_2_) across the entire body, is determined by measuring whole-body VO_2_ during incremental exercise. According to the Fick principle, VO_2_ is determined by cardiac output and arteriovenous oxygen difference. During incremental exercise, VO_2_ is strongly influenced by arteriovenous oxygen difference, reflecting the oxidative capacity in active muscles^9–11^. Muscle oxygen saturation (SmO_2_), an indicator of skeletal muscle oxygenation, offers partial insights into the oxidative capacity in active muscles since SmO_2_ reflects the balance of oxygen demand and supply in the tissue^12–14^. Furthermore, several previous studies have noted that individuals exhibiting a greater decrease in SmO_2_ in active muscles during incremental exercise tend to demonstrate higher peak VO_2_^12,15–17^. Therefore, focusing on skeletal muscle oxygenation is crucial for enhancing exercise tolerance. Nevertheless, the characteristics of individuals exhibiting a greater decrease in SmO_2_ in active muscles during incremental exercise remain elusive. A previous study suggested that the reduction in SmO_2_ is more pronounced in individuals achieving higher peak work rates during incremental exercise^18^. In essence, this decline in SmO_2_ is greater in those attaining longer peak exercise durations during incremental exercise, where the work rate increases steadily at a constant rate per minute^18–20^. Consequently, individuals demonstrating a greater decrease in SmO_2_ throughout incremental exercise are presumed to possess the capacity for prolonged muscle contraction, signifying elevated muscle endurance.

Furthermore, strong muscle contractions become imperative beyond the midpoint of incremental exercise, owing to the continual increase in work rate at a steady rate per minute, as previously mentioned^18–20^. Consequently, individuals with robust muscle endurance solely at low intensities might encounter challenges in maintaining muscle contraction post the midpoint of incremental exercise. Therefore, possessing substantial muscle endurance at moderate-to-high intensities becomes indispensable for sustaining muscle contraction throughout incremental exercise.

The intensity of muscle endurance is strongly influenced by muscle strength due to the measurement method^21^. Typically, muscle endurance is quantitatively assessed by evaluating the rate of decrease relative to maximum muscle strength^21^. Consequently, individuals with higher muscle strength tend to exhibit higher intensity of muscle endurance. Based on this premise, we hypothesized that individuals with moderate-to-high levels of both muscle strength and endurance would experience a greater decrease in SmO_2_ during incremental exercise. Therefore, this study aimed to elucidate the relationship among muscle strength, muscle endurance, and skeletal muscle oxygenation dynamics in active leg muscles during incremental exercise.

## Methods

### Participants and an overview of the experimental protocol

This study enrolled healthy young men aged 18–30 years. Considering the known differences in skeletal muscle oxygenation dynamics between men and women^13^, we specifically enrolled men. Participants were recruited through research subject recruitment posters and social media platforms. A total of 29 healthy young men participated in the study, each involved for over two separate days. Muscle strength and endurance assessments were conducted on the first day, whereas cardiopulmonary exercise testing (CPX) combined with near-infrared spectroscopy (NIRS) was performed on the second day. Participants were instructed to abstain from consuming caffeine, eating, or smoking for at least 2 h before and during the tests. Of the initial 29 participants, three were excluded due to a peak respiratory exchange rate (RER) of < 1.1 during CPX, one due to inaccurate measurements caused by equipment defects, and one due to contracting COVID-19 during the study period. Consequently, 24 healthy young men were included in the final analysis. These participants were further categorized into two groups based on their muscle strength and endurance levels: the non-moderate-to-high muscular strength and endurance group (characterized by low leg muscle strength, endurance, or both) and the moderate-to-high muscular strength and endurance group (characterized by moderate-to-high levels of both muscle strength and endurance). Before enrollment, all participants were thoroughly briefed on the research objectives and potential risks, and they provided informed consent. The study protocol adhered to the principles outlined in the Declaration of Helsinki and received approval from the Ethics Committee of Showa University (Protocol No.: 22-193-A; Approval date: November 9, 2022).

### Participant’s basic characteristics

We assessed the age, height, body mass (BM), BM index, body fat rate, leg adipose mass, and exercise habits of all participants on the first or second day. Additionally, we measured body fat percentage and leg adipose mass using a bioelectrical impedance analyzer (InBody 470, InBody Japan Inc., Japan). To evaluate exercise habits, participants were verbally asked if they engage in sports or other leisure activities at least once a week.

### Muscle strength and endurance

Leg muscle strength and endurance of the right leg were sequentially measured, with a 10-min rest between the two assessments, using an isokinetic dynamometer (Biodex System 4, Biodex Medical System Inc., Shirley, New York, USA) for all participants. Peak torque during knee extension served as the measure of leg muscle strength. In this study, peak torque was defined as the maximum torque produced during five consecutive muscle contractions performed at an angular velocity of 60°/s^22^. It was measured twice with a 2-min rest between sets, and the highest value of peak torque was divided by BM to obtain the analytical value. Regarding leg muscle endurance, we evaluated the strength decrement index (SDI) during knee extension^21^. SDI was defined as the percentage decrease in the average value of the last 10 torques relative to the maximum peak torque during 30 consecutive muscle contractions performed at an angular velocity of 180°/s. Therefore, SDI was calculated using the following formula:

1 $${\text{SDI }} = \, \left( {{\text{maximum peak torque }} - {\text{ average value of last 1}}0{\text{ torques}}} \right) \, /{\text{ maximum peak torque }} \times \, 100$$

We measured the SDI once and used that value as the analytical value. Prior to strength and muscular endurance testing, all participants underwent a single movement verification.

**Figure 1:**
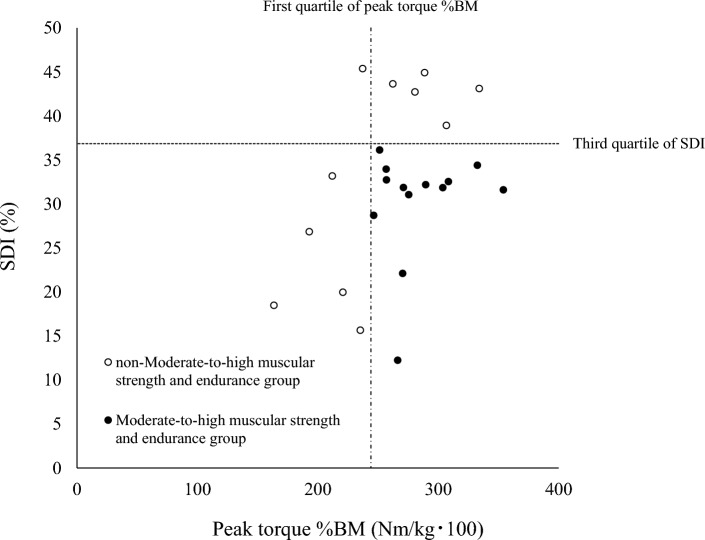
Relationship between leg muscle strength and leg muscle endurance in all participants and participants by group. Black and white circles represent the correlation between leg muscle strength and endurance in the moderate-to-high muscular strength and endurance group and the non-moderate-to-high muscular strength and endurance group, respectively.

In this study, a peak torque value below the first quartile indicated low muscle strength, whereas a value above the first quartile indicated moderate-to-high muscle strength, as higher peak torque values reflect greater muscle strength. Similarly, an SDI value above the third quartile indicated low muscle endurance, whereas a value below the third quartile indicated moderate-to-high muscle endurance, as higher SDI values generally indicate lower muscle endurance (Fig. 1).

### Whole-body oxygen uptake, RER, and external load during incremental exercise

We utilized CPX to measure whole-body VO_2_ during incremental exercise, employing cycling ergometers (Rehcor 500P, Lode, Netherlands) for the procedure. Participants were seated on the saddle of the cycling ergometers and rested for 3 min. Following a 3-min warm-up at 10 W, participants engaged in exercise with a progressively increasing load, incrementing by 20 W every minute. They were instructed to maintain a pedal rotation speed of 60 revolutions per minute throughout the exercise^23^. For risk management during CPX, we continuously monitored participants’ heart rate using electrocardiography (Life Scope 8, Nihon Kohden Co., Japan) and blood pressure using a blood pressure cuff and an automatic blood pressure monitor (EBP-330, Minato Medical Science Co., Ltd., Japan) at 1-min intervals. Concurrently, we continuously measured VO_2_ and carbon dioxide production using an expired gas analyzer (Aero Monitor AE-310 s, Minato Medical Science Co., Ltd., Japan) during CPX. RER was calculated as the ratio of carbon dioxide production to VO_2_. Expired gas was sampled using the breath-by-breath method. Calibrations of the gas meter and transducer were performed according to the manufacturer’s instructions before each test. In CPX, the incremental exercise was terminated when participants could not maintain 50 revolutions per minute^23^. We considered a participant to have exerted maximal effort during CPX if the RER value exceeded 1.1. The highest points of VO_2_, RER, and work rate during incremental exercise were regarded as their peak values. VO_2_ and RER were averaged over 9 breaths, whereas work rate was based on a single time point to determine the peak values. The duration from the start to the end of the incremental exercise during CPX represented the exercise time.

### Skeletal muscle oxygenation and blood volume during incremental exercise

Leg muscle oxygenation and muscle blood volume were continuously measured using a NIRS device (MOXY^®^, Fortiori Design LLC, Hutchinson, Minnesota, USA) during CPX^12^. The MOXY monitor emits light waves (630–850 nm) sequentially from four light-emitting diodes into the tissue beneath the device and records the amount of scattered light returned by two detectors located at 12.5 and 25 mm from the light source^12^. This mechanism enables the MOXY monitor to locally measure muscle oxygen saturation (SmO_2_) and total hemoglobin (THb) by interpreting hemoglobin and myoglobin oxygen saturation in muscle capillaries. Further details on the measurement principle can be found in a previous study^12^. This device has demonstrated reliability and validity in previous studies^12,24,25^. In the present study, changes in SmO_2_ and THb of the right lateral vastus lateralis reflected leg muscle oxygenation dynamics and muscle blood volume dynamics, respectively^12^. To determine the SmO_2_ and THb of the right lateral vastus lateralis, we positioned the MOXY monitor 5 cm lateral to the midpoint of the imaginary line between the upper edge of the patella and the greater trochanter of the right femur^12,26–28^. We secured this monitor to the measurement site with adhesive tape after attaching the supplied light shield according to the manufacturer’s instructions. The mean value of SmO_2_ during the 3-min resting period served as the SmO_2_ value at rest, whereas the value at 10 s into each stage of the incremental exercise represented the SmO_2_ value at each stage. Based on these values, we calculated the difference in SmO_2_ between rest and each stage and the change in SmO_2_ from rest to each stage using the following formula to assess leg muscle oxygenation dynamics at each stage:

2 $$Difference{\text{-SmO}_{2}}\text{-}each\, stage \left( \% \right) \, = {\text{ value of SmO}}_{{2}} {\mathrm{\,at\, rest\, {-} \,value\, of\, SmO}}_{{2}} {\mathrm{\,at\, each\, stage}}$$

3 $$Change{\text{-SmO}_{2}}{\text-}{each\,stage}\, ({\%}) = \frac{(\mathrm{value\, of\, Sm}{{\text{O}}}_{2}\mathrm{ \,at \,rest } - \mathrm{ value\, of\, Sm}{{\text{O}}}_{2}\mathrm{ \,at \,each \,stage}) }{\mathrm{ value\, of \,Sm}{{\text{O}}}_{2}\mathrm{ \,at\, rest}} \times 100$$

Similarly, for changes in muscle blood volume dynamics, the difference in THb between rest and each stage and the change in THb from rest to each stage were determined in the same manner as the SmO_2_ values.

### Statistical analysis

Results are presented as mean ± SD or median (interquartile range) for participant characteristics data. The normality of distribution was assessed using the Anderson–Darling test and histogram statistics. Differences in participant characteristics between the non-moderate-to-high muscular strength and endurance group and the moderate-to-high muscular strength and endurance group were analyzed using the unpaired t-test, Mann–Whitney *U*-test, and Chi-square test. Additionally, effect sizes for the unpaired t-test and Mann–Whitney *U*-test were calculated. Multiple regression analyses, employing the forced-entry method, were conducted to examine relevant factors influencing leg muscle oxygenation dynamics. Differences in changes of leg muscle oxygenation dynamics and muscle blood volume dynamics during incremental exercise between the two participant groups were analyzed using the linear mixed-effect model and the Holm method for multiple-comparison tests. In cases where the linear mixed-effect model indicated a significant interaction, the Holm method was utilized. The relationships among muscle strength, endurance, and leg muscle oxygenation dynamics, as well as between leg muscle oxygenation dynamics and peak VO_2_, were assessed using Pearson’s product–moment correlation coefficient. A P-value of < 0.05 was considered statistically significant. All statistical analyses were performed using JMP^®^ Pro 15 (SAS Institute Inc., Cary, NC, USA). Prior to the study, a power analysis for linear mixed-effects models for repeated measures was conducted using a sample size determination tool^29,30^. Based on a previous study^19^, the sample size was calculated to be 11 in each group (total of 22) for the linear mixed-effects model assessing differences in changes of leg muscle oxygenation dynamics (standard deviation of group A = 6.3; standard deviation of group B = 5.5; mean difference = 7.6; α error probability = 0.05; power [1 − β error probability] = 0.8). Therefore, the sample size in the present study was considered sufficient for employing linear mixed-effects models.

## Results

Of the 24 participants, 11 were assigned to the non-moderate-to-high muscular strength and endurance group and 13 to the moderate-to-high muscular strength and endurance group. In the non-moderate-to-high muscular strength and endurance group, five participants had moderate-to-high muscle strength only (with low muscle endurance), five had moderate-to-high muscle endurance only (with low muscle strength), and one had both low muscle strength and low muscle endurance (Fig. 1 and Table 1). A significant positive correlation was observed in all participants between peak torque %BM and SDI (r = 0.411, *P* = 0.046). Table 1 shows the differences in participant characteristics between the non-moderate-to-high muscular strength and endurance group and the moderate-to-high muscular strength and endurance group. Peak VO_2_, peak VO_2_/BM, peak work rate, peak work rate/BM, exercise time during CPX, and exercise habit were significantly lower in the non-moderate-to-high muscular strength and endurance group than in the moderate-to-high muscular strength and endurance group (*P* = 0.019, *P* = 0.005, *P* = 0.007, *P* = 0.007, *P* = 0.007, and *P* = 0.028, respectively). Regarding exercise habits, the non-moderate-to-high muscular strength and endurance group comprised two participants solely engaged in strength training, whereas the moderate-to-high muscular strength and endurance group included two participants with both strength and endurance training habits, three participants involved in soccer, one participant in ice hockey, one participant in high-intensity cycling, and one participant with other habits. No significant difference was observed in muscle strength and endurance between the two groups.

**Table 1:** Participant characteristics.

	Non-moderate-to-high muscular strength and endurance group (n = 11)	Moderate-to-high muscular strength and endurance group (n = 13)	*P*-value	Effect size
Age (years)	22.0 (21.0–24.0)	21.0 (19.5–22.0)	0.089	*r* = 0.35
Height (cm)	171.3 ± 4.6	171.0 ± 4.1	0.879	*d* = 0.06
BM (kg)	62.5 (60.6–78.9)	61.2 (59.0–66.9)	0.385	*r* = 0.18
BMI (kg/m^2^)	21.9 (20.9–25.5)	21.4 (20.4–23.3)	0.354	*r* = 0.19
Body fat rate (%)	18.6 ± 7.0	16.5 ± 7.2	0.482	*d* = 0.29
Dominant foot (right/left)	11/0	12/1	0.261	–
Right leg adipose mass (kg)	2.2 ± 1.1	1.9 ± 0.9	0.508	*d* = 0.28
Right leg adipose mass %BM (kg/kg･100)	3.0 ± 0.9	2.9 ± 1.0	0.702	*d* = 0.16
Peak torque (Nm)	168.1 ± 31.8	179.2 ± 18.9	0.301	*d* = 0.43
Peak torque %BM (Nm/kg･100)	248.5 ± 51.3	283.3 ± 32.9	0.057	*d* = 0.82
SDI (%)	38.9 (20.0–43.7)	31.9 (29.9–33.4)	0.224	*r* = 0.25
Moderate-to-high muscle strength and endurance	0	13	–	–
Moderate-to-high muscle strength (with low muscle endurance)	5	0	–	–
Moderate-to-high muscle endurance (with low muscle strength)	5	0	–	–
Low muscular strength and endurance	1	0	–	–
Peak VO_2_ (mL/min)	2381.5 ± 234.6	2782.4 ± 476.8	0.019	*d* = 1.04
Peak VO_2_/BM (mL/kg/min)	35.3 ± 5.5	43.9 ± 7.7	0.005	*d* = 1.27
Peak respiratory exchange rate	1.23 (1.18–1.26)	1.19 (1.16–1.25)	0.310	*r* = 0.21
Peak work rate (W)	208.2 ± 15.9	240.0 ± 32.5	0.007	*d* = 1.21
Peak work rate/BM (W/kg)	3.1 ± 0.5	3.8 ± 0.6	0.007	*d* = 1.06
Exercise time during CPX (s)	594.3 ± 47.2	690.0 ± 97.6	0.007	*d* = 1.20
Exercise habit (yes/no)	2/9	8/5	0.028	–

Values are expressed as mean ± SD or median (interquartile range).

Differences in participant characteristics between the moderate-to-high muscular strength and endurance group and the non-moderate-to-high muscular strength and endurance group were analyzed using the unpaired *t*-test, Mann–Whitney *U*-test, and Chi-square test.

*BM* body mass, *BMI* body mass index, *SDI* strength decrement index, *VO*_*2*_ oxygen uptake, *CPX* cardiopulmonary exercise test.

Tables 2 and 3 show the relevant factors influencing leg muscle oxygenation dynamics in all participants. The groups (1; non-moderate-to-high muscular strength and endurance group, 2; moderate-to-high muscular strength and endurance group) and the *difference-THb-100%peak VO*_*2*_ were entered into a multiple regression model for predicting *difference-SmO*_*2*_*-100%peak VO*_*2*_ using the forced-entry method in all participants. The moderate-to-high muscular strength and endurance group significantly predicted the *difference-SmO*_*2*_*-100%peak VO*_*2*_ in all participants (β = 0.59, *P* = 0.002) (Table 2). Furthermore, the groups (1; non-moderate-to-high muscular strength and endurance group, 2; moderate-to-high muscular strength and endurance group) and *change-THb-100%peak VO*_*2*_ were entered into a multiple regression model for predicting *change-SmO*_*2*_*-100%peak VO*_*2*_ using the forced-entry method in all participants. We found that the moderate-to-high muscular strength and endurance group significantly predicted the *change-SmO*_*2*_*-100%peak VO*_*2*_ in all participants (β = 0.56, *P* = 0.006) (Table 3).

**Table 2:** Relevant factors influencing leg muscle oxygenation dynamics (difference in SmO_2_ between rest and each stage) in all participants.

Independent variables	Dependent variable: *difference*-SmO_2_-*100%peak VO*_*2*_				
	B ± SE	β	95% CI of B	SSE	*P*-value
Moderate-to-high muscular strength and endurance group	8.0 ± 2.3	0.59	3.3 to 12.7	1746.7	0.002
*Difference*-THb-*100%peak VO*_*2*_ (%)	− 15.8 ± 11.9	− 0.22	− 40.4 to 8.9	206.5	0.198
Constant	18.8 ± 2.9	0.00	12.8 to 24.8	–	< 0.001
	Coefficient of determination R^2^ = 0.443, F = 8.342, *P* = 0.002				

The group (1; non-moderate-to-high muscular strength and endurance group, 2; moderate-to-high muscular strength and endurance group) and the *difference*-THb-*100%peak VO*_*2*_ (%) were entered into a multiple regression model for predicting relevant factors of the *difference*-SmO_2_-*100%peak VO*_*2*_ using the forced-entry method.

*B* partial regression coefficient, *β* standardized partial regression coefficient, *SE* standard error, *CI* confidence interval, *SSE* sum of squared errors.

**Table 3:** Relevant factors influencing leg muscle oxygenation dynamics (change in SmO_2_ from rest to each stage) in all participants.

Independent variables	Dependent variable: *change*-SmO_2_-*100%peak VO*_*2*_				
	B ± SE	β	95% CI of B	SSE	*P*-value
Moderate-to-high muscular strength and endurance group	14.0 ± 4.6	0.56	4.5 to 23.5	4857.1	0.006
*Change*-THb-*100%peak VO*_*2*_ (%)	− 1.1 ± 3.1	− 0.07	− 7.5 to 5.2	64.7	0.717
Constant	30.8 ± 5.9	0.00	18.6 to 43.0	–	< 0.001
	Coefficient of determination R^2^ = 0.328, F = 5.119, *P* = 0.016				

The group (1; non-moderate-to-high muscular strength and endurance group, 2; moderate-to-high muscular strength and endurance group) and the *change*-THb-*100%peak VO*_*2*_ (%) were entered into a multiple regression model for predicting relevant factors of the *change*-SmO_2_-*100%peak VO*_*2*_ using the forced-entry method.

*B* partial regression coefficient, *β* standardized partial regression coefficient, *SE* standard error, *CI* confidence interval, *SSE* sum of squared errors.

**Figure 2:**
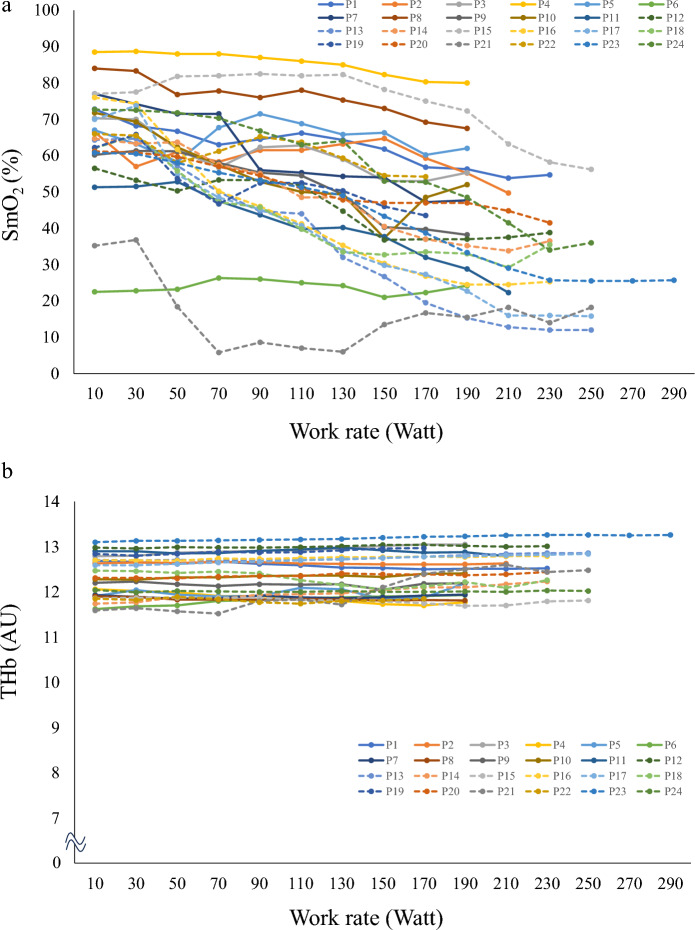
Individual raw NIRS traces during incremental exercise. Solid and dotted lines represent individual raw NIRS traces from the non-moderate-to-high muscular strength and endurance group and the moderate-to-high muscular strength and endurance group, respectively. (**a**) Individual raw SmO_2_ traces from all participants. (**b**) Individual raw THb traces from all participants. *P* participant.

Figure 2 displays individual raw NIRS traces during incremental exercise. Two participants completed the incremental exercise test at < 190 watts. Conversely, the participant with the longest exercise duration during the test completed the incremental exercise at approximately 290 watts.

**Figure 3:**
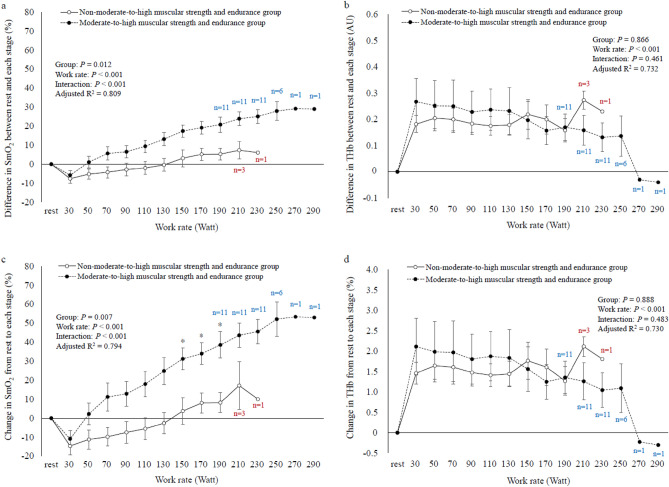
Differences in the change in leg muscle oxygenation dynamics or muscle blood volume dynamics during incremental exercise between the non-moderate-to-high muscular strength and endurance group and the moderate-to-high muscular strength and endurance group (exercise load represented by work rate). Error bars represent standard error (SE). Black and white circles represent the change in leg muscle oxygenation dynamics or muscle blood volume dynamics during incremental exercise in the moderate-to-high muscular strength and endurance group and the non-moderate-to-high muscular strength and endurance group, respectively. Because only one participant in the non-moderate-to-high muscular strength and endurance group was able to continue exercising until 230watts, the range of the mixed model was from rest to 210watts, and the range of multiple comparisons was from 30 to 210watts. Blue letters indicate the number of participants in the moderate-to-high muscular strength and endurance group and red letters indicate the number of participants in the non-moderate-to-high muscular strength and endurance group at each stage. Asterisk denotes a significant difference between the two groups at each stage (*P* < 0.05). (**a**) Differences in *difference*-SmO_2_-*each stage* between the two groups during incremental exercise. (**b**) Differences in the *difference*-THb-*each stage* between the two groups during incremental exercise. (**c**) Differences in the *change*-SmO_2_-*each stage* between the two groups during incremental exercise. (**d**) Differences in the *change*-THb-*each stage* between the two groups during incremental exercise.

Figure 3 shows the differences in leg muscle oxygenation dynamics or muscle blood volume dynamics during incremental exercise between the two participant groups. In the linear mixed-effect model with the *difference*-SmO_2_-*each stage* as the dependent variable, individual participants as random effects, and group and work rate as fixed effects (adjusted R^2^ = 0.809), significant main effects were observed in group and work rate (*P* = 0.012 and *P* < 0.001, respectively), with a significant interaction between them (*P* < 0.001). However, multiple-comparison test results showed no significant difference in the *difference*-SmO_2_-*each stage* between the two groups (Fig. 3a). In the linear mixed-effect model with the *difference*-THb-*each stage* as the dependent variable, individual participants as random effects, and group and work rate as fixed effects (adjusted R^2^ = 0.732), a significant main effect was noted in work rate alone (*P* < 0.001), with no significant interaction between group and work rate (Fig. 3b). Moreover, in the linear mixed-effect model with the *change*-SmO_2_-*each stage* as the dependent variable, individual participants as random effects, and group and work rate as fixed effects (adjusted R^2^ = 0.794), significant main effects were observed in group and work rate (*P* = 0.007 and *P* < 0.001, respectively), with a significant interaction between them (*P* < 0.001). Multiple-comparison test results showed that the *change*-SmO_2_-*150Watt*–*190Watt* was significantly higher in the moderate-to-high muscular strength and endurance group than in the non-moderate-to-high muscular strength and endurance group (Fig. 3c). In the linear mixed-effect model with the *change*-THb-*each stage* as the dependent variable, individual participants as random effects, and group and work rate as fixed effects (adjusted R^2^ = 0.730), a significant main effect was noted in work rate alone (*P* < 0.001), with no significant interaction between group and work rate (Fig. 3d).

**Figure 4:**
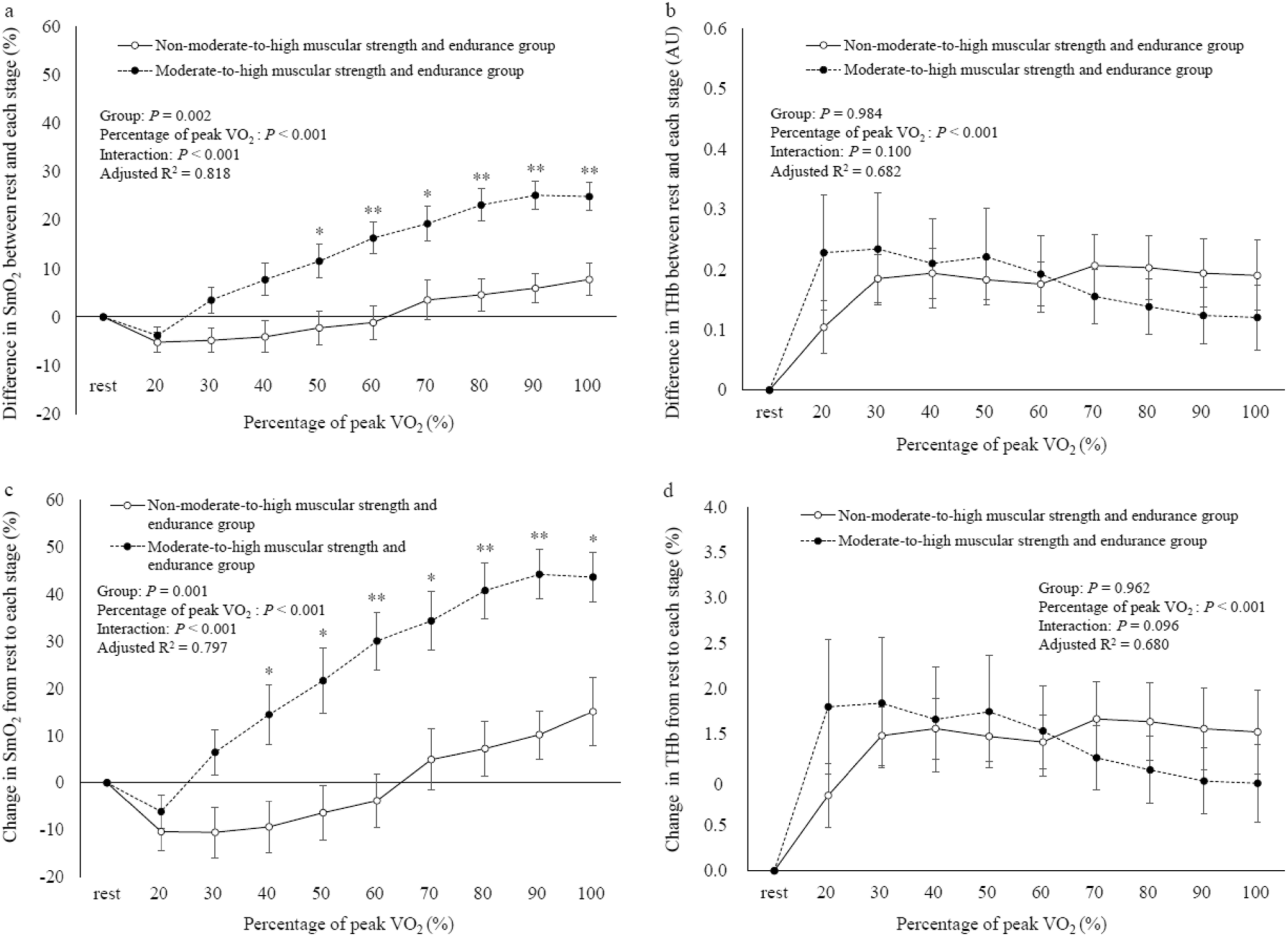
Differences in the change in leg muscle oxygenation dynamics or muscle blood volume dynamics during incremental exercise between the non-moderate-to-high muscular strength and endurance group and the moderate-to-high muscular strength and endurance group (exercise load represented by percentage of peak VO_2_). Error bars represent standard error (SE). Black and white circles represent the change in leg muscle oxygenation dynamics or muscle blood volume dynamics during incremental exercise in the moderate-to-high muscular strength and endurance group and the non-moderate-to-high muscular strength and endurance group, respectively. Asterisk and double asterisk denote a significant difference between the two groups at each stage (*P* < 0.05 and *P* < 0.01, respectively). (**a**) Differences in *difference*-SmO_2_-*each stage* between the two groups during incremental exercise. (**b**) Differences in the *difference*-THb-*each stage* between the two groups during incremental exercise. (**c**) Differences in the *change*-SmO_2_-*each stage* between the two groups during incremental exercise. (**d**) Differences in the *change*-THb-*each stage* between the two groups during incremental exercise.

Figure 4 displays the differences in the change in leg muscle oxygenation dynamics or muscle blood volume dynamics during incremental exercise between the two participant groups. Using a linear mixed-effects model with the *difference*-SmO_2_-*each stage* as the dependent variable, individual participants as random effects, and group and percentage of peak VO_2_ as fixed effects (adjusted R^2^ = 0.818), significant main effects were observed for group and percentage of peak VO_2_ (*P* = 0.002 and *P* < 0.001, respectively), with a significant interaction between them (*P* < 0.001). Furthermore, multiple-comparison test results revealed that the difference in SmO_2_*-50–100%peak VO*_*2*_ was significantly higher in the moderate-to-high muscular strength and endurance group than in the non-moderate-to-high muscular strength and endurance group (Fig. 4a). Similarly, in the linear mixed-effects model with the *difference*-THb-*each stage* as the dependent variable, individual participants as random effects, and group and percentage of peak VO_2_ as fixed effects (adjusted R^2^ = 0.682), a significant main effect was observed for percentage of peak VO_2_ alone (*P* < 0.001), with no significant interaction between group and percentage of peak VO_2_ (Fig. 4b). Moreover, analyzing the linear mixed-effects model with the *change*-SmO_2_-*each stage* as the dependent variable, individual participants as random effects, and group and percentage of peak VO_2_ as fixed effects (adjusted R^2^ = 0.797), significant main effects were observed for group and percentage of peak VO_2_ (*P* = 0.001 and *P* < 0.001, respectively), with a significant interaction between them (*P* < 0.001). Multiple-comparison test results showed that the *change*-SmO_2_-*40–100%peak VO*_*2*_ was significantly higher in the moderate-to-high muscular strength and endurance group than in the non-moderate-to-high muscular strength and endurance group (Fig. 4c). In the linear mixed-effects model with the *change*-THb-*each stage* as the dependent variable, individual participants as random effects, and group and percentage of peak VO_2_ as fixed effects (adjusted R^2^ = 0.680), a significant main effect was observed for percentage of peak VO_2_ alone (*P* < 0.001), with no significant interaction between group and percentage of peak VO_2_ (Fig. 4d).

**Figure 5:**
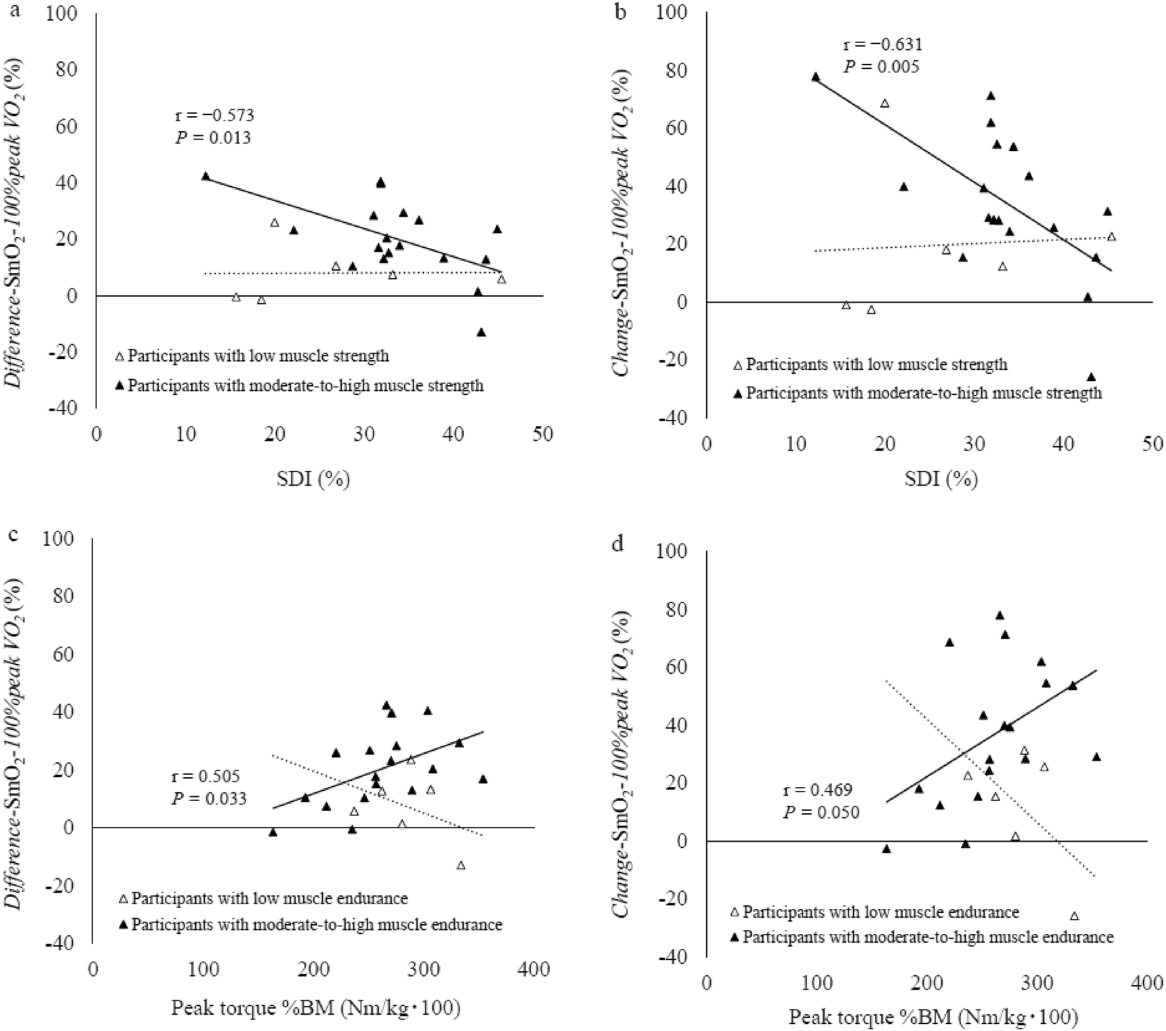
Relationship among muscle strength, muscle endurance, and leg muscle oxygenation dynamics. In (**a,b**), black and white triangles represent the correlation between leg muscle oxygenation dynamics at peak exercise load and muscle endurance in participants with moderate-to-high muscle strength and low muscle strength, respectively. Approximately straight lines with a solid black line depict the same correlation in participants with moderate-to-high muscle strength, whereas approximately straight lines with a black dotted line represent this correlation in participants with low muscle strength. In (**c,d**), black and white triangles represent the correlation between leg muscle oxygenation dynamics at peak exercise load and muscle strength in participants with moderate-to-high muscle endurance and low muscle endurance, respectively. Approximately straight lines with a solid black line depict the same correlation in participants with moderate-to-high muscle endurance, whereas approximately straight lines with a black dotted line represent this correlation in participants with low muscle endurance.

Figure 5 shows the relationship among muscle strength, muscle endurance, and leg muscle oxygenation dynamics. Although the *difference*-SmO_2_-*100%peak VO*_*2*_ and *change*-SmO_2_-*100%peak VO*_*2*_ were not significantly correlated with SDI in all participants and participants with low muscle strength, a significantly negative correlation was observed between the *difference*-SmO_2_-*100%peak VO*_*2*_ and SDI and between the *change*-SmO_2_-*100%peak VO*_*2*_ and SDI in participants with moderate-to-high muscle strength (r =  − 0.573, *P* = 0.013 and r =  − 0.631, *P* = 0.005, respectively) (Fig. 5a and 5b). The *difference*-SmO_2_-*100%peak VO*_*2*_ and *change*-SmO_2_-*100%peak VO*_*2*_ also were not significantly correlated with peak torque %BM in all participants and participants with low muscle endurance; however, we noted significantly positive correlations between the *difference*-SmO_2_-*100%peak VO*_*2*_ and peak torque %BM and between the *change*-SmO_2_-*100%peak VO*_*2*_ and peak torque %BM in participants with moderate-to-high muscle endurance (r = 0.505, *P* = 0.033 and r = 0.469, *P* = 0.050, respectively) (Fig. 5c and 5d).

Moreover, the *difference*-SmO_2_-*100%peak VO*_*2*_ and *change*-SmO_2_-*100%peak VO*_*2*_ were significantly positively correlated with peak VO_2_/BM in all participants (r = 0.551, *P* = 0.005 and r = 0.582, *P* = 0.003, respectively).

## Discussion

Given that exercise tolerance is a strong predictor of life prognosis regardless of health condition^1–4^, it is imperative for individuals to enhance exercise tolerance by increasing daily physical activity. The oxidative capacity in active muscles strongly influences exercise tolerance^9–11^, with skeletal muscle oxygenation dynamics providing partial information about this capacity^12–14^. Therefore, although it is crucial to focus on skeletal muscle oxygenation dynamics to improve exercise tolerance, the characteristics of individuals with high skeletal muscle oxygenation dynamics in active muscles during incremental exercise remain unclear. In this study, we investigated the relationship among muscle strength, muscle endurance, and skeletal muscle oxygenation dynamics in active leg muscles during incremental exercise. Our results revealed that participants with both moderate-to-high leg muscle strength and endurance exhibited higher muscle oxygenation dynamics at moderate- or high-intensity loads compared to those with low leg muscle strength, endurance, or both, regardless of blood volume dynamics. Furthermore, leg muscle oxygenation dynamics at high-intensity loads was positively correlated with muscle endurance in participants with moderate-to-high leg muscle strength and positively correlated with muscle strength in those with moderate-to-high leg muscle endurance.

### Differences in participant characteristics between the two groups

In this study, no significant differences were observed in muscle strength and muscle endurance between the two groups. This lack of difference may be attributed to an inverse correlation between these factors. In the moderate-to-high muscular strength and endurance group, many participants displayed moderate values for both muscle strength and muscle endurance. Conversely, in the non-moderate-to-high muscular strength and endurance group, many participants exhibited high values for either muscle strength (but low values for muscle endurance) or high values for muscle endurance (but low values for muscle strength). Consequently, upon averaging muscle strength and muscle endurance values in each group, no significant differences were observed between the two groups.

Additionally, no significant differences were found in right leg adipose mass and right leg adipose mass %BM between the two groups. As for adipose tissue thickness (ATT) of the right lateral vastus lateralis, this parameter was not assessed in the study. Previous studies have indicated that ATT scatters the NIRS signal before reaching the muscle^31^, and the MOXY device is also influenced by ATT^24,25^. Therefore, measuring ATT and adjusting for its effects in future studies is essential for clarifying our results.

### Relationship among muscle strength, muscle endurance, and muscle oxygenation dynamics

In the present study, leg muscle oxygenation dynamics during moderate- or high-intensity loads were higher in participants with moderate-to-high muscle strength and endurance than in those with low muscle strength, endurance, or both. Given that SDI is calculated as the rate of decrease relative to the maximum torque^21^, participants with moderate-to-high muscle strength and endurance were deemed to possess the muscle endurance necessary to sustain moderate or higher muscle power. Conversely, participants with low muscle strength, endurance, or both lacked the muscle endurance required to sustain moderate or higher muscle power (although the latter group comprised participants with high muscular endurance, they only had enough muscle endurance to sustain low muscle power due to their low muscle strength) Sustaining moderate or higher muscle power necessitates a high percentage of type I and type IIA muscle fibers. Type I fibers have a slow twitch but are relatively fatigue-resistant due to their high oxidative and low glycolytic capacities, whereas type IIA fibers have a fast and strong twitch and are relatively fatigue-resistant due to their high oxidative and glycolytic capacities^32–34^. Therefore, participants with moderate-to-high muscle strength and muscle endurance in our study were presumed to possess a high percentage of type I and type IIA muscle fibers. Endurance training alone or in combination with strength training increases the percentage of type I and type IIA muscle fibers^35–37^. In our study, many participants with moderate-to-high muscle strength and endurance engaged in various forms of active exercise, such as strength training, endurance training, soccer, ice hockey, and high-intensity cycling. This observation suggests that individuals with moderate-to-high muscle strength and endurance tend to have a higher percentage of type I and type IIA muscle fibers. Furthermore, a previous study demonstrated a positive association between the percentage of type I muscle fibers and peak VO_2_, as well as between type IIA muscle fibers and muscle strength and endurance^38^. Given that participants with moderate-to-high muscle strength and endurance exhibited higher peak VO_2_ and good muscle strength and endurance, we hypothesized that these participants likely have a higher percentage of both type I and type IIA muscle fibers.

During incremental exercise, type I muscle fibers, characterized by a slow twitch and relative fatigue resistance, primarily engage under moderate intensity loads, whereas type IIA muscle fibers, known for their fast twitch and resistance to fatigue, primarily activate under high-intensity loads. Additionally, these muscle fiber types possess high muscle oxidative capacity during exercise due to abundant mitochondria and high capillary density^35^. Consequently, because participants with moderate-to-high muscle strength and endurance were presumed to have a high percentage of both type I and type IIA muscle fibers, leg muscle oxygenation dynamics at moderate- or higher-intensity loads were greater in these participants than in those with low muscle strength, endurance, or both in the present study.

### Training application

Our study findings suggest that maintaining moderate-to-high levels of both muscle strength and endurance contributes to enhanced muscle oxygenation dynamics during moderate- or higher-intensity exercise. Whole-body VO_2_ during physical activity is influenced by the muscle oxidative capacity of active muscles and cardiac output^9–11^. Therefore, when active muscles possess high oxidative capacity during the same load activity, it alleviates the cardiac workload. Because skeletal muscle oxygenation dynamics provides partial information about the oxidative capacity in active muscles^12–14^, maintaining moderate-to-high levels of both muscle strength and endurance may be beneficial for individuals with low cardiac function, such as older adults and patients with cardiac problems. Additionally, we found that leg muscle oxygenation dynamics at high-intensity loads was positively correlated with leg muscle endurance only in participants without low leg muscle strength and with leg muscle strength only in those without low leg muscle endurance. The former result may be attributed to an overestimation of muscle endurance when muscle strength is low, as SDI was calculated as the rate of decrease relative to the maximum torque. Thus, a positive relationship was not observed between muscle oxygenation dynamics and muscle endurance in participants with low leg muscle strength. The latter result may be attributed to lower muscle oxygenation dynamics at lower muscle endurance, even with high muscle strength, due to a weak negative relationship between leg muscle strength and leg muscle endurance. Some participants had both high muscle strength and low muscle endurance. Therefore, this study also indicates that increasing both muscle strength and endurance contributes to enhancing muscle oxygenation dynamics, and achieving this through exercise training is crucial. Conversely, combining strength and endurance training has been reported to impair muscle strength development compared to strength training alone^39^. However, this finding was derived from a previous study with notably high volume, frequency, and intensity. In another study, combining resistance and endurance training improved muscle strength and endurance capacity in untrained men when the training frequency and intensity were moderate^40^. Furthermore, adding endurance training to resistance training improved muscle oxygen capacity without negative consequences for muscle strength in older and younger individuals undergoing strength training^41^. Thus, despite the relatively low negative relationship between muscle strength and muscle endurance in the present study, exercise training has the potential to enhance both muscle strength and endurance.

### Limitations

This study has several limitations. First, we could not evaluate ATT, and consequently, the results of this study were not adjusted for ATT. Assessing and adjusting for ATT in future investigations will be important to better understand its impact on study outcomes. Second, we could not examine muscle metabolism during incremental exercise because blood lactate levels were not evaluated. Thus, evaluating blood lactate levels in future research is necessary. Third, due to the small sample size and cross-sectional design, we could not confirm whether the improvement of both leg muscle strength and endurance contributes to the skeletal muscle oxygenation of active leg muscles during incremental exercise and adjust for several participant characteristics, such as exercise habits and exercise tolerance. Therefore, a large-scale interventional study should be conducted in the future. Finally, our study findings may not be applicable to women, older adults, and patients with cardiac problems. Consequently, conducting an interventional study including these populations is necessary to verify the present study results.

## Conclusions

Participants with moderate-to-high levels of both muscle strength and endurance exhibited higher skeletal muscle oxygenation dynamics (i.e., greater decrease in SmO_2_) in the leg muscles during moderate- or high-intensity exercise loads compared to those with low levels of muscle strength, muscle endurance, or both. Therefore, increasing both muscle strength and endurance is necessary to enhance skeletal muscle oxygenation dynamics during incremental exercise.

## Data Availability

The datasets generated during and/or analyzed during the current study are available from the corresponding author on reasonable request.
